# Duration of Prebiotic Intake Is a Key-Factor for Diet-Induced Modulation of Immunity and Fecal Fermentation Products in Dogs

**DOI:** 10.3390/microorganisms8121916

**Published:** 2020-12-02

**Authors:** Mariana P. Perini, Mariana F. Rentas, Raquel Pedreira, Andressa R. Amaral, Rafael V. A. Zafalon, Roberta B. A. Rodrigues, Lucas B. F. Henríquez, Lucca Zanini, Thiago H. A. Vendramini, Júlio C. C. Balieiro, Cristiana F. F. Pontieri, Marcio A. Brunetto

**Affiliations:** 1 Pet Nutrology Research Center, Nutrition and Animal Production Department, School of Veterinary Medicine and Animal Science (FMVZ), University of São Paulo (USP), Pirassununga, SP 13635-900, Brazil; mariana.perini@usp.br (M.P.P.); mariana.rentas@usp.br (M.F.R.); rafael_rafa_az@hotmail.com (R.V.A.Z.) roberta_barodrigues@hotmail.com (R.B.A.R.); lucas.henriquez@usp.br (L.B.F.H.); lucca.zanini@usp.br (L.Z.); thiago.vendramini@usp.br (T.H.A.V.); balieiro@usp.br (J.C.C.B.); 2 Nutritional Development Center, Grandfood Industry and Commerce LTD (Premier Pet), Dourado, SP 13590-000, Brazil; rpedreira@premierpet.com.br (R.P.); cristiana@premierpet.com.br (C.F.F.P.); 3 Veterinary Nutrology Service, Teaching Veterinary Hospital, School of Veterinary Medicine and Animal Science (FMVZ), University of São Paulo (USP), São Paulo, SP 05508-010, Brazil; andressa.rodrigues.amaral@usp.br

**Keywords:** nutrient, gut health, immunity, prebiotics

## Abstract

Prebiotics promote health benefits, however, there is no consensus on the minimal intake period required in order to obtain good results. This study evaluated the effect of the time of ingestion of prebiotics on fecal fermentation products and immunological features in dogs. Twenty-four adult dogs were randomly distributed in a block design with six groups and four treatments. Diet and intake period were variation factors. Diets were either a control diet without the addition of prebiotic (CO) or with the inclusion of 1% of a commercial product containing a minimum of 0.38% galactooligosaccharides (GOS), 0.5% (B1) or 1% (B2) of a prebiotic blend. Time variable was set at 30 and 60 days for evaluation of immunity and gut health. Results were analyzed in the Statistical Analysis System software (SAS), version 9.4, considering the repeated measures over time design, and means were compared by the Tukey test and *p* < 0.05 was significant. Propionic acid was the only variable that had an interaction effect, with reduction of this metabolite in treatment B2 in the period of 60 days. At T60, concentrations of immunoglobulin A, lactic acid, and pH in the feces increased (*p* < 0.05) in all treatments regardless of prebiotic inclusion or not. GOS increased fecal score and lactic acid concentrations. Therefore, a 60-day intake period of a prebiotic blend was not sufficient to modulate fecal and immune variables and higher concentrations of a single prebiotic would be more relevant for results.

## 1. Introduction

Prebiotics are defined as substrates that are not digestible by the animal’s digestive enzymes and are only fermented by beneficial microbiota of the large intestine, improving the host’s overall health. The growth of beneficial microbiota enhances the production of microbiota metabolic products such as short-chain fatty acids (SCFA), branched-chain fatty acids (BCFA), and lactic acid [1,2]. In addition, the effects of prebiotics are not limited to the intestine alone, but can also provide systemic effects such as modulation of the immune system [3]. The most commonly used prebiotics in diet formulations for dogs and cats are fructooligosaccharides (FOS), mananoligosaccharides (MOS) [4,5,6], yeast cell wall (YCW) [7], and β-glucans [8,9]. Galactooligosaccharides (GOS) are poorly studied in dogs and only one study included this product in their diets [10]. However, studies have shown that the use of a prebiotic blend can modulate more effects than a single prebiotic [11,12,13,14]. 

It is now known that the goal of dog nutrition is not only satiety and survival, but also well-being, health improvement, and disease attenuation [15]. Researchers as well as nutritionists in the pet food industry often look for functional ingredients that offer benefits beyond basic nutrition [16]. The duration of prebiotic intake required in order to obtain these benefits is not yet defined and encourages further studies on the subject. According to [17], there is no consensus on the minimal time required to observe the effects of foods in dogs and cats. Most studies (39% of 23 studies) with prebiotic interventions used a shorter period, on average 14 days [7,10,11,12,13,14,18,19,20] with few benefits that would justify its inclusion. On the other hand, 26% of the studies evaluated different prebiotics for an average of 24 days [21,22,23,24,25,26] and 13% evaluated for 33 days on average [27,28,29]. About 13% evaluated the long-term effects during 220 days on average [30,31,32]. Finally, the minority (9%) of studies evaluated an average of 79 days [16,33]. Based on the information collected, it can be concluded that there is no consensus regarding the minimum ideal time for evaluating the effects of prebiotics in commercial pet food, and this knowledge is of great importance in understanding their effects in a diet [34].

Therefore, this study aimed to evaluate the time of ingestion of different prebiotics on the concentrations of fermentative products in the feces and local (intestinal) immunity of adult dogs.

## 2. Materials and Methods 

This study was previously approved by the Ethics Committee of Veterinary Medicine and Animal Science School of the University of São Paulo (FMVZ/USP) under the protocol 8591050419.

### 2.1. Animals, Location, and Experimental Design

Twenty-four healthy adult dogs (males and females) of different breeds with a mean age of 6.0 ± 2.22 years and an ideal body condition score (BCS) according to [35] were selected. The animals were housed in individual boxes (2 × 5.60 m) with bed and solarium (2 × 4.90 m) and water was provided ad libitum. The dogs were released for physical activity and socialization for six hours a day when they were not in the collection period. This animals were distributed in a randomized block design and divided into six groups (according to their metabolic weight) with four treatments: control diet without the addition of prebiotics (CO); control diet with addition of 1% of a commercial product composed of at least 0.38% galactooligosaccharides (GOS); control diet with the addition of 0.5% commercial Blend Yes Golf^®^ (B1); and control diet with the addition of 1% commercial Blend Yes Golf^®^ (B2). Commercial blends consisted of a mixture of at least 120 g/kg of FOS, 60 g/kg of MOS, 72 g/kg of GOS, organic zinc, and 150 g/kg of 1,3 β-glucans. The GOS treatment contained at least 380 g/kg of galactooligosaccharides.

The animals were fed twice daily. The amount of food was set according to the maintenance energy requirement (MER) predicted with the equation: MER-95 × (body weight)^0.75^ [17]. All dogs were weighed weekly for possible adjustments in the amount of food offered in order to avoid energy imbalance. The leftovers were also weighed and the amount of food consumed was recorded for later consumption calculation.

The study length was 60 days and divided into two periods of 30 days each, T30 (30 days) and T60 (60 days), for comparisons. The first 20 days were destined for diet adaptation, then five days was destined to the evaluation of fecal score (FS), two days for rest, and the last three days for feces collections and evaluation of fecal pH, SCFA (propionate, butyrate, acetate), total SCFA, BCFA, total BCFA, lactic acid, ammonia nitrogen, and immunoglobulin A (IgA).

Both the extrusion of experimental diets and the experimental periods itself were carried out at Grandfood Industry (Premier pet), located in the city of Dourado, São Paulo, Brazil. Foods were formulated according to [36] and prebiotics were added to the topping. 

The chemical composition of the experimental diets is shown in Table 1.

### 2.2. Feces Analysis (FS, pH, SCFAs, BCFAs, NH_3_, Lactic Acid, and IgA)

Fecal score was determined according to [37] on a scale ranging from one to five and classified according to the feces characteristics: 1 = hard, dry and crumbly; 1.5 = hard and dry; 2 = firm but not hard that leaves little or no residue on ground when picked up; 2.5 = firm, moist surface, leaves residue but holds form when picked up; 3 = wet but with no form when picked up; 3.5 = very wet with no form when picked up; 4 = no defined shape and very wet; 4.5 = watery but with some pieces with shape; and 5 = watery, no shape. After FS was recorded, feces were collected within 15 min after defecation, homogenized with a vortex homogenizer at room temperature, and weighed. 

The pH was assessed with a digital bench gauge (Digimed, DM-20, Quimis do Brasil Ltd. A., São Paulo, Brazil) after the electrode was introduced in a diluted solution of 9:1 of distilled water and feces. The pH was determined according to the methodology adapted from [38]. This analysis was carried out at the Premier Pet Nutritional Development Center in Dourado, São Paulo.

Analysis of SCFAs and BCFAs were carried out at the Ruminal Fermentability Laboratory of the Food Engineering and Animal Science School of the University of São Paulo (ZAZ/FZEA USP). Three grams of fresh feces, up to a maximum of 15 min after defecation, were acidified with 9 mL of 16% formic acid. The mixture was kept in a refrigerator at 4 °C for seven days, homogenized daily in a vortex spinning machine at room temperature, and later centrifuged for 15 min at 15 °C and 5000 rpm, using the supernatant and discarding the sediment three times in a row. After extraction, the samples were identified and stored at −15 °C. The determination of SCFAs and BCFAs were performed by gas chromatography (Shimadzu gas chromatography GC-2014, Shimadzu of Brazil, São Paulo-SP, Brazil) according to the methodology in [39] with a flame ionization detector control by the Shimadzu GC Solution program, and a Stalbilwax 30 m × 0.53 mm separation column, helium gas as the carrier, nitrogen as make up and manual regulation, flame detector at 250 °C, and temperature inside the column kept at 145 °C. For calibration, external standard solution with acetic, propionic, butyric, isobutyric, valeric, and isovaleric acid was used. 

The ammoniacal nitrogen (NH_3_) analysis was carried out at the Multi-User Laboratory of Animal Nutrition and Bromatology of the Department of Animal Nutrition and Production of FMVZ/USP where 3 g of feces were acidified with 9 mL of 16% formic acid. The mixture was centrifuged at 5000 rpm for 15 min, at 15 °C three times, using the supernatant and discarding the sediment. After extraction, the samples were identified and stored at −15 °C until analysis. The extracts were thawed at room temperature and then 2 mL aliquots were diluted in 13 mL of distilled water and analyzed in the nitrogen distiller. Distillation was performed with 5 mL of 2N potassium hydroxide solution and titration with 0.005 mol/L of hydrochloric acid, according to the methodology in [40].

For lactic acid analysis, 2 g of fresh feces were mixed with 6 mL of distilled water (1:2 w/v) and samples were quantified according to [41] by the spectrophotometry method at 565 nm (500 to 570 nm), in which a white reagent was used in order to calibrate the spectrophotometer (QUICK-Lab, DRAKE Eletronica Commercia LTD, São José do Rio Preto, Brazil). The samples were quantified by comparing them with 0.08% lactic acid standard.

Finally, for the quantification of IgA in feces, 3 g of fresh sample was immediately stored at −20 °C. After thawing, IgA was extracted with saline according to the methodology in [42]. Reading was performed on an ELISA Microplate Reader (Microplate Reader MRX TC Plus, Dynex Technologies, Chantilly, VA, USA) using a 450 nm filter. These analyzes were carried out at a laboratory specialized in scientific analysis (LEAC) in São Paulo/SP.

### 2.3. Statistical Analysis

The data were processed with Statistical Analysis System software, version 9.4 (SAS Institute Inc., Cary, NC, USA). The normality of the residuals was verified by the Shapiro-Wilk test using the univariate procedure from SAS and the homogeneity of the variances by the Levine’s test. All variables were analyzed using a mixed model that included the fixed effects of treatment (CO, GOS, B1 and B2), time (T30 and T60), and treatment versus time interaction, in addition to the random effects of blocks and residue. All analyses were performed using the PROC MIXED procedure. The fixed effect model of treatments remained, in addition to the random effect of the residue. 

In the case of significant ANOVA statistics, the averages were compared by the Tukey test at the level of 5% significance, according to the following statistical model:

Y_ij_ = µ + t_i_ + b_k_ + p_j_ + tp_ik_ + e_ijk_ (1)

where Y_ij_ is the dependent variable; µ is the overall mean; t_i_ is the fixed treatment effect; b_j_ is the random block effect; pk is the fixed time effect; t_i_p_k_ is the fixed effect of treatment x time interaction; and e_ijk_ is the residual error. 

## 3. Results

**Figure 1:**
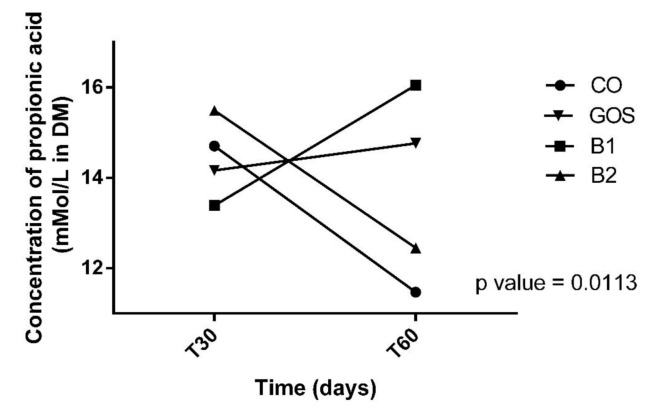
Effect of treatment x time interaction of propionic acid. CO: control food, without the addition of prebiotic; GOS: control food with addition of 0.38% galactooligosaccharides; B1: control food with addition of 0.5% YES GOLF^®^ prebiotic blend; B2: control food with the addition of 1% YES GOLF^®^ prebiotic blend; T30: 30-day period; T60: 60-day period.

**Figure 2:**
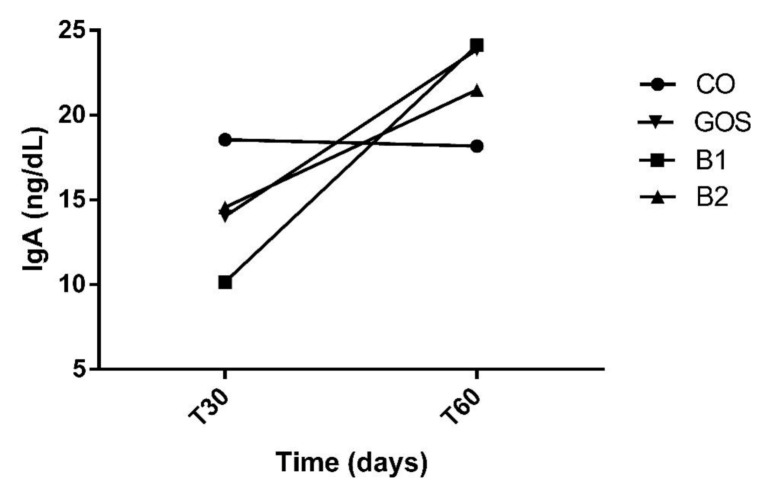
Increased fecal IgA at T60. Despite having no effects, at T60, IgA increased in CO, GOS, and B1 treatments. CO: control food, without the addition of prebiotic; GOS: control food with addition of 0.38% galactooligosaccharides; B1: control food with addition of 0.5% YES GOLF^®^ prebiotic blend; B2: control food with the addition of 1% YES GOLF^®^ prebiotic blend; T30: 30-day period; T60: 60-day period.

All animals had an adequate food intake, therefore, the addition of the prebiotic to the diet did not affect palatability. There was also no food rejection or diarrhea. The analysis of variances on the effects of time, treatment, and interaction are shown in Table 2. Propionic acid was the only variable with significant changes in treatment by period interaction (*p* = 0.0113, Figure 1). Propionic acid was reduced in treatment B2 at T60, while in other treatments, this effect was not observed (Figure 1 and Table 2). In Figure 1, this variable increased in B1 at T60, but this result was not significant. The products of fecal fermentation and immunological variables such as lactic acid, pH, and IgA (Figure 2) had a time effect at T60 as observed with the increase in their concentrations. The other variables showed no difference (*p* > 0.05) in both the treatment factor and the time factor, except for the fecal score and lactic acid, which were increased in dogs receiving GOS supplementation compared to the CO and B1 treatments. 

## 4. Discussion

This was the first study that showed the effect of time on immunological and gut health features in dogs consuming prebiotics. The reduction in propionic acid can be explained according to the concentration of the prebiotic and intestinal microbiota. Another study demonstrated a reduction in propionate in dogs supplemented with 66% pectin and 33% of cellulose, and 100% pectin [43]. Despite the reduction found, our dogs did not have diarrhea and fecal scores were within the ideal range. 

The potential of a prebiotic can be assessed according to fermentation products formed such as propionic acid [44], that is, the B2 treatment was the worse prebiotic blend for this variable. Despite this small reduction, studies [12] have shown that SCFA can be absorbed into the colon by colonocytes, which affect the fecal concentrations of these fermentative products. As SCFAs are volatile, there is a loss in their concentration within the sample during processing. However, in our study, we acidified the feces with formic acid and this caused the SCFA to be retained in the solution, which prevents evaporation and the aforementioned loss. This metabolite has an anti-inflammatory effect on human colon cancer cells [45]. In dogs and cats, the function of propionic acid is not yet fully elucidated, however, it is known that this is a gluconeogenic SCFA that can be converted to glucose via succinyl-CoA and the oxaloacetate route [46,47,48]. In addition, propionic acid can stimulate the absorption of fluids, calcium, magnesium, and other cations in the colon [49]. Intermediate fermentation products such as lactic, succinic, pyruvic acid, and ethanol are usually metabolized to SCFA, but when produced in large quantities, they can be excreted in the feces [50]. 

The longest time of ingestion (60 days) changed fecal pH, lactic acid, and IgA for all treatments. Studies with fish also showed that fish fed with 1, 8, and 15 g.kg^−1^ of MOS (into the water) [51] or 0.05%, 0.10% and 0.20% (of dry matter) of MOS in fish food [52] for 45 and 60 days, respectively, had their lactic acid bacteria concentrations increased. In the present study, the increase in fecal pH was an unexpected finding, since the increase in lactic acid is generally associated with a reduction in fecal pH [3,53]. However, this increase in fecal pH corroborates the results found in some studies that evaluated the inclusion of prebiotics in dogs in a period of 14 and 28 days [11,26]. The authors [11] observed a tendency to increase fecal pH in cannulated dogs fed with low or high crude protein plus a 1.5 g FOS/kg supplementation for a short period of 14 days. Another study [26] also observed an increase in the pH of feces in the 28 days. The slight increase in lactic acid concentrations and the non-increase of SCFA are possible explanations for not reducing fecal pH. 

The increase in fecal IgA is corroborated in studies in rodents [54]. The study in [54] found effects in 42 days of experimentation. Another study in dogs also showed a tendency to increase the concentration of IgA, when the animals were fed for a period of 14 days [7]. The mechanisms involved in the actions of prebiotics in modulating immunological variables in the intestine have not yet been fully elucidated [55]. According to [53], the inclusion of the prebiotic as well as the time of use and its source can influence its effect. 

The intestinal microbial modulation is the target effect of prebiotics, and this still requires further studies since there is no consensus about the time required for a prebiotic to modulate the microbiota and result in the expected effects. In our study, the duration of intake was able to modulate some interesting aspects. The concentration of fecal IgA increased with the addition of prebiotics over time, which is an interesting result that shows the improvement of the local immunological capacity after a longer period of exposure to the prebiotic. Other studies have observed variations in fecal IgA concentrations in dogs over time [56,57]. The continuous production of IgA occurs due to constant stimulation of the intestinal microbiota [58]. Beneficial bacteria stimulate the immune system, which produces mediators (cytokines, mononuclear cells, and immunoglobulins) that interact with the immune system controlling it. Each prebiotic works in different ways, however, the pathways for microbiota and immune system modulation are not fully elucidated. FOS changes the intestinal environment that produces favorable conditions for the production of secretory IgA; in addition, its administration may increase the blood concentrations of IL-10 and IFN-γ [54]. In addition to the positive regulation of mucosal immune response, FOS is also a substrate for *Lactobacillus spp.* and *Bifidobacterium spp.* growth, both responsible for lactic acid, SCFA, and IgA production [59]. Studies have shown that FOS stimulated the production of CD4 and CD8 cells, which indicates that it could act as an immunostimulatory agent in endotoxemia and, therefore improve immunocompetence [60,61]. 

On the other hand, MOS supplementation acts differently from other prebiotics, since this substrate prevents the invading bacteria from binding in the gut mucosa, acting as a blocker [16]. In addition to directly influencing the population of bacteria, MOS can increase the activity of lysozymes, antibodies [52], and CD4 + T cells, which indicates that this supplementation may have an influence on the immune humoral response [32]. The combination of FOS and MOS can increase local levels and systemic immune characteristics [12]. The GOS prebiotic contains structures similar to those found in microvilli membranes that interfere with the bacterial receptor and therefore prevent harmful bacteria from binding to the epithelium. In addition, GOS (6% of the diet) increased the secretion of intestinal and fecal IgA in mice and humans [62,63].

The inclusion of different prebiotics did not alter the concentrations of SCFA, total SCFA, total BCFA, and ammonia nitrogen, except for propionic acid regarding time and treatment effect, which corroborated the results of [12] and [19]. The study in [12] mentioned that the lack of alteration might have occurred due to the high absorption of SCFAs by colonocytes, positively affecting microbial populations without affecting the fecal concentrations.

However, other studies that have used higher levels of prebiotics (from 1 to 3 g/day and 15 g/kg of diet) showed an increase in SCFA concentrations [23,28,32] and the higher concentration of the prebiotic may have an impact on a different result. The unchanged concentration of BCFA was expected, since most studies have found no effect for this variable [11,12,13,18,19,21,26,27,29,33,64]. 

Regarding ammoniacal nitrogen, studies have shown that the inclusion of 1 g/kg of body weight of transoligosaccharides, 5 g/L diluted inulin, and 0.3%, 0.6%, and 0.9% of inulin and oligofructose resulted in its reduction [11,33,65]. The concentrations of ammoniacal nitrogen are extremely relevant because in high concentrations, they can cause damage to health in dogs and cats, which causes a reduction in the height of the villi and a decrease in the absorption of nutrients after its absorption in the intestinal wall [66,67].

As for the treatment effect, GOS increased the concentration of lactic acid as well as the results obtained by [67], who used 0.3% and 0.6% of inactive yeast Saccharomyces cerevisiae for 37 days. GOS can influence the increase in lactic acid concentrations since it stimulates the proliferation of lactic acid bacteria and *Bifidobacterium spp*, which is responsible for producing it; however, this does not mean that the concentrations of propionic acid will increase since there are absorptions of this metabolite in the colon [65]. A study with sheep with the inclusion of 20 g/kg BW^0.75^ of GOS influenced the increase in lactic acid [68]. According to [50], lactic acid concentrations increased in elderly humans supplemented with 5.5 g/day of GOS. 

Regarding to fecal characteristics, its consistency is one of the most important variables when evaluating dog foods [22]. In the present study, the fecal score was influenced (increased) only by GOS, with a value of 2.8 (above the ideal), according to [37]. Despite the FS value being above the ideal range, the animals did not present diarrhea or fecal malformation, which indicates that the fecal form was still ideal and that the inclusion of GOS is safe. There was no effect on the treatment by time interaction and the time of ingestion of different prebiotics in the FS. According to the only study that used 1 g/kg BW/d of transgalactooligosaccharides (TGOS) in the feeding of dogs, the FS was within the ideal range (3.5) [10]. The concentration of the prebiotic and the concentration of SCFA are important factors in the quality and fecal production, since high concentrations of this contribute to the increase of humidity in the feces and consequently contributes to the increase in the fecal score [17]. 

According to the results of the study, the time of 60 days had a positive influence on some variables, however, the period did not modulate the effects of treatments B1 and B2, maybe because the concentrations of prebiotic mixtures were low and the time to modulate them should be bigger. There are no minimal period recommendations for checking the effects on a dog or cat food [17]. Studies that used higher concentrations of prebiotic blends (2.5% in the diet) observed beneficial effects over a period of 84 days [13]. The use of an isolated prebiotic in high concentrations (≥1.0%) can be considered as the best option compared to a mixture with low concentrations (<1%) of each [12,14,22]. In addition, studies that took ≥60 days observed beneficial effects with concentrations (<1%) of prebiotics [13,58,69,70]. Studies that used ≤40 days had positive effects with high concentrations (≥1%) [10,14,21,22,26]. That is, for concentrations below 1% of prebiotics, the minimum period should be 60 days.

## 5. Conclusions

The 60-day period can be considered safe in the inclusion of different prebiotics in adult dogs. The longer time of ingestion of prebiotics contributed to increased immunity and improved fecal fermentation products. Additionally, providing the prebiotic alone with an adequate concentration must be taken into consideration. In addition, the GOS treatment increased the concentration of lactic acid and fecal score, which indicates that the inclusion of this prebiotic can be considered safe for adult dogs. 

## Figures and Tables

**Table 1:** Chemical composition of the experimental diets.

Item	Treatments			
%	CO ^1^	GOS ^2^	B1 ^3^	B2 ^4^
Dry matter	91.95	91.80	91.76	91.76
Ash	6.47	6.61	6.72	6.85
Crude protein	27.46	26.93	25.28	27.22
Fat	14.17	14.53	14.32	14.37
Crude fiber	6.30	6.40	5.97	5.83
Nitrogen-free extract	45.60	45.53	47.71	45.73

^1^ CO: control group (diet without addition of prebiotics); ^2^ GOS: food with the addition of 0.38% galactooligosaccharides (min. 380 g/kg); ^3^ B1: food with the addition of 0.5% Blend Yes Golf^®^; ^4^ B2: food with addition of 1% of Blend Yes Golf^®^.

**Table 2:** Average concentrations of fecal and immunological fermentative products of adult dogs fed with different prebiotics.

Item	CO	GOS	B1	B2		MTE		*p*		
	Averages				SD	Average	SD	Treat.	Time	Treat. × Time
**Latic acid (mMol/L in solution)**										
**T30**	2.13	2.15	2.12	2.14		2.13 ^B^				
**T60**	2.16	2.19	2.16	2.18	0.007	2.17 ^A^	0.0037	0.0002	<0.0001	0.7515
**MPE**	2.14 ^b^	2.17 ^a^	2.14 ^b^	2.16 ^a,b^	0.005					
**NH_3_ (mMol/kg in DM)**										
**T30**	119.87	133.58	157.58	140.7		137.93				
**T60**	145.73	123.32	136.90	136.61	14.75	135.64	8.7738	0.5565	0.8144	0.3775
**MPE**	132.80	128.45	147.24	138.66	11.13					
**pH**										
**T30**	6.75	6.77	6.65	6.58		6.69 ^B^				
**T60**	6.98	6.96	6.92	6.76	0.11	6.91 ^A^	0.0681	0.2004	0.0043	0.9696
**MPE**	6.87	6.87	6.78	6.67	0.08					
**Acetic acid (mMol/L in DM)**										
**T30**	22.50	19.98	19.15	22.23		22.23				
**T60**	18.24	26.38	23.61	19.23	2.42	19.23	2.4202	0.6638	0.6029	0.0836
**MPE**	20.37	23.18	21.38	20.73	1.71					
**Propionic acid (mMol/L in DM)**										
**T30**	14.70	14.16	13.38	15.49 ^A^		14.43				
**T60**	11.47	14.76	16.05	12.45 ^B^	1.38	13.68	0.9051	0.7222	0.2664	0.0113
**MPE**	13.09	14.46	14.72	13.97	1.09					
**Butyric acid (mMol/L in DM)**										
**T30**	4.20	3.62	4.87	4.47		4.29				
**T60**	4.04	4.46	4.06	4.63	0.50	4.30	0.25	0.6934	0.9847	0.4473
**MPE**	4.12	4.04	4.46	4.55	0.35					
**Total SCFA (mMol/L in DM)**										
**T30**	41.41	37.78	37.41	42.20		39.70				
**T60**	33.76	45.61	43.72	36.32	3.95	39.85	2.11	0.7406	0.9555	0.1085
**MPE**	37.59	41.69	40.57	39.26	2.86					
**Iso-butyric (mMol/L in DM)**										
**T30**	0.76	0.79	0.85	0.82		0.81				
**T60**	0.76	0.78	0.88	0.84	0.10	0.82	0.050	0.7104	0.9033	0.9958
**MPE**	0.76	0.79	0.87	0.83	0.07					
**Iso-valeric (mMol/L in DM)**										
**T30**	0.95	0.80	1.14	0.85		0.93				
**T60**	1.12	0.85	0.96	0.94	0.10	0.97	0.052	0.0892	0.6463	0.3716
**MPE**	1.04	0.82	1.05	0.89	0.07					
**Valeric (mMol/L in DM)**										
**T30**	0.12	0.12	0.11	0.10		0.11				
**T60**	0.14	0.13	0.11	0.09	0.01	0.12	0.008	0.0934	0.5187	0.6574
**MPE**	0.13	0.13	0.11	0.09	0.01					
**Total BCFA (mMol/L in DM)**										
**T30**	1.82	1.72	2.15	1.78		1.87				
**T60**	2.00	1.75	2.01	1.84	0.19	1.90	0.090	0.3164	0.8144	0.8594
**MPE**	1.91	1.73	2.08	1.81	0.13					
**IgA (ng/mL)**										
**T30**	14.03	10.15	14.57	18.55		14.33 ^B^				
**T60**	23.87	24.11	21.49	18.18	4.80	21.91 ^A^	3.008	0.9796	0.0182	0.4167
**MPE**	18.95	17.13	18.03	18.37	3.70					
**Fecal score**										
**T30**	2.55	2.85	2.55	2.51		2.61				
**T60**	2.50	2.78	2.50	2.51	0.10	2.57	0.057	0.0124	0.5664	0.9891
**MPE**	2.52 ^b^	2.81 ^a^	2.52 ^b^	2.51 ^b^	0.07					

DM: dry matter; CO: control food, without the addition of prebiotic; GOS: control food with addition of 0.38% galactooligosaccharides; B1: control food with addition of 0.5% YES GOLF^®^ prebiotic blend; B2: control food with the addition of 1% YES GOLF^®^ prebiotic blend; MTE: Main treatment effect; T30: 30-day period; T60: 60-day period; MPE: Main period effect; ^A; B^ Averages in the same column and followed by the same capital letter, do not differ at 5% by the Tukey Test; ^a; b^ Averages in the same line and followed by the same lower case letter do not differ at 5% by the Tukey Test.
